# Concurrent cervical and extra cervical HPV-related lesions: an observational study

**DOI:** 10.1007/s10096-026-05423-6

**Published:** 2026-04-29

**Authors:** I. Palaia, E. N. Cavallari, F. Tanzi, A. Pierangeli, M. Ralli, P. G. Meliante, I. Clementi, V. Di Donato, G. Perniola, A. Pernazza, G. Palaia, Investigators of the H. P. V. Unit, U. Romeo, M. De Vincentiis, L. Muzii, C. Della Rocca, G. D’Ettorre, C. M. Mastroianni, A. Polimeni, G. Antonelli, G. Bogani, S. Mancini, G. Gentile

**Affiliations:** 1 Department of Gynaecology, Maternal and Child Health and Urological Sciences, Sapienza University of Rome, Rome, Italy; 2 Department of Public Health and Infectious Diseases, Sapienza University of Rome, Rome, Italy; 3 Department of Molecular Medicine, Laboratory of Virology, Sapienza University of Rome, Rome, Italy; 4 Department of Sense Organs, Sapienza University of Rome, Rome, Italy; 5 Department of Emergency, Sapienza University of Rome, Rome, Italy; 6 Department of Radiological, Oncological and Pathological Sciences, Sapienza University of Rome, Rome, Italy; 7 Department of Oral and Maxillofacial Sciences, Sapienza University of Rome, Rome, Italy

**Keywords:** HPV, Cervical HPV infection, Anal HPV infection, Oral HPV infection, Otorhinolaryngologic HPV infection

## Abstract

**Purpose:**

This prospective observational study aims to determine the prevalence of concurrent Human Papillomavirus (HPV) infections in different anatomical sites in patients with a cervical HPV (cHPV) infection.

**Methods:**

Patients with cHPV infection referred to the Gynecological HPV Unit of Policlinico Umberto I, Sapienza, University of Rome were recruited. Inclusion criteria were: women ≥ 25 and < 80 years old; positive cHPV-DNA test; signed informed consent. The exclusion criteria were: (I) pregnancy; (II) previously treated cancer in the investigated sites (cervical, oral and anal districts); and (III) any cancer under active treatment. All enrolled patients underwent gynecological examination, Pap-smear, colposcopy and, if appropriate, biopsies. Recruited patients were addressed to proctological (High Resolution Anoscopy-HRA/ HPV DNA/ Pap smear), otorhinolaryngological (laryngoscopy and biopsy when appropriate) and odontostomatological evaluation (clinical examination and biopsy when appropriate) in the corresponding Departments of Policlinico Umberto I involved in the multidisciplinary unit.

**Results:**

From March 2022 to December 2023, 150 patients were enrolled in the study. The cervical Pap test was abnormal in 101 patients (67.3%); 46 patients (30.5%) reported a cervical intraepithelial neoplasia Grade 2 + (CIN2 +) after cervical biopsies. Anal HPV (aHPV) test was positive in 77 patients of 150 (51.3%), 93 patients underwent HRA reporting anal high grade squamous intraepithelial lesion (aHSIL) in 39% and anal low grade squamous intraepithelial lesion (aLSIL) in 46.9% of 64 patients subjected to biopsies. Eighty-one women (54%) refused odontostomatological examination. HPV related oral and laryngeal lesions were detected in 8 of 69 and 10 of 150 patients respectively. Cervical/anal coinfection was frequent (67%) and mostly observed in CIN2 + patients (p < 0.05), regardless of age, immunosuppressive conditions or smoking habits. Cervical/otorhinolaryngological and cervical/odontostomatological coinfection were rare (6.7% and 2.9% respectively).

**Conclusions:**

In CIN2 + patients the risk of aHPV coinfection is non-negligible; these findings prompt us to consider anal brushing in these patients. On the other hand, data regarding oral and laryngeal HPV lesions do not support its routine evaluation. Future and larger studies are needed to consolidate these hypotheses of our exploratory study, paving the way to multidisciplinary guidelines for future practice.

## Introduction

In recent years, few pathogens have garnered as much scientific and public health attention as Human Papillomavirus (HPV), which constitutes the most prevalent sexually transmitted infection globally. With over 200 genotypes identified, HPV is responsible for more than 14 million new infections annually, and it is estimated that over 80% of the population will acquire the virus at some point during their lifetime [1].

Given the global incidence of HPV-related diseases, recent research has raised concerns about emerging concomitant infections across multiple anatomical sites [2]. These infections can result in a wide range of clinical manifestations, from asymptomatic cases to benign conditions (such as papilloma and condylomas), and even to the development of various cancers. The progression and outcome depend on factors such as the infected anatomical site, immune status, microbiome composition, and viral genotype.

The overall burden of HPV-related cancers has been estimated to be approximately 5% of all human cancers, with 610,000 cases/year worldwide, 50,000 of which occur in Europe [3]. Among these, high-risk HPV genotypes (HR-HPV) account for more than 90% of cervical cancer cases, around 90% of anal cancers, 50–60% of oropharyngeal squamous cells carcinomas, 5% of oral cancers and a minority of cases of vulvar, vaginal and penile cancers [4].

Currently, anti HPV-vaccination together with screening programs for the prevention of cervical cancer play a key role in reducing the burden of HPV infection and its associated neoplastic disease [5].

On the other hand, although the incidence of HPV related anal and oropharyngeal cancer is increasing, the application of extra-gynaecological screening programs is still debated, due to the low incidence of anal/oral related cancers, together with the lack of shared multidisciplinary guidelines and the absence of facilitated pathways for patients affected.

Considering the challenges in preventing and managing multi-site HPV infections and the associated risk of HPV-related neoplasia, the main outcome of this prospective, observational, multidisciplinary study is to quantify the prevalence of concomitant extra-cervical HPV infections; the secondary outcome was to define the clinical and pathological characteristics of extra-cervical HPV lesions and examine their relationship with the clinical and histopathological profile of cervical disease.

## Material and methods

### Study design

This is a prospective observational multidisciplinary study that involved the departments of Gynaecology, Infectious Disease, Proctology, Otorhinolaryngology, Dentistry, Virology and Pathology of Policlinico Umberto I Hospital, Sapienza University of Rome.

The protocol was approved by the institutional ethics committee (Protocol Number: 0166/2022, Rif. 6593). Data was stored in an appropriate shared database.

### Case selection

From March 2022 to December 2023 patients with a positive cervical HPV (cHPV) test attending the gynaecologic outpatient clinic of Policlinico Umberto I of Rome were consecutively enrolled in the study. Inclusion criteria were: (I) women with age ≥ 25 and < 80 years; (II) positive cHPV-DNA test. The exclusion criteria were: (I) pregnancy; (II) previously treated cancer in the investigated sites (cervical, oral and anal districts); and (III) any cancer under active treatment. All participants signed a written informed consent.

### Study procedures and outcomes

Patients enrolled, beside the management of the cHPV infection and related conditions, were submitted to anal, odontostomatologic and otorhinolaryngologic evaluation. The outcomes of each district-specific investigation were collected by a single operator (F.T.), while the other operators involved were blinded to the results.

Data regarding HPV vaccination, smoking habits, parity, presence of immunosuppressive conditions, and use of concomitant estrogen-progestin therapy were collected in a shared database.

#### Cervical screening

Patients with a positive cHPV DNA test underwent Pap smear and/or colposcopy following International Guidelines [6]; colposcopic findings were classified accordingly as normal, Atypical Transformation Zone type 1 (ANTZ1), or type 2 (ANTZ2).

Pap smears were classified according to the 2015 Bethesda System [7] in Negative, Atypical Squamous Cells of Undetermined Significance (ASCUS), Low Grade Squamous Intraepithelial Lesions (LSIL) and High Grade Squamous Intraepithelial Lesions (HSIL).

Targeted biopsies were performed in case of suspicious lesions at colposcopy [6]. Histopathological classification of cervical lesions was performed according to the World Health Organization (WHO) Classification of Female Genital Tumours (2020) [8] and the terminology was aligned with the recommendations of the Lower Anogenital Squamous Terminology (LAST) Project [9] as follows: Cervical Intraepithelial Neoplasia Grade 1 (CIN1), grade 2 (CIN2), grade 3 (CIN3) and Carcinoma in Situ.

Patients in whom histologically confirmed LSIL—CIN1 was found were referred to follow-up with Pap smear and colposcopy after six months. Patients with HSIL—CIN2/3 underwent conization surgery, preferably by Loop Electrosurgical Excision Procedure (LEEP). Non-vaccinated patients were invited to get a nonavalent vaccine immediately before or after the treatment.

#### Anal examination

All participants underwent anal swabs for HPV DNA detection and genotyping and anal cytology.

High resolution anoscopy (HRA) was offered to participants and was conducted according to current guidelines on practice standards for the detection of anal cancer precursors [10]. Targeted biopsies of anal mucosa and/or perianal skin were collected from suspicious lesions. Histopathological classification of anal lesions was performed according to the recommendations of the Lower Anogenital Squamous Terminology (LAST) Project [9] as follows: LSIL and HSIL.

#### Odontostomatological examination

The odontostomatological clinical evaluation consists of a traditional physical examination: the health of the oral cavity was analysed, focusing on the evaluation of the oral mucosa, to detect any suspicious HPV-related lesions. After the dental examination, patients were classified as disease-free if no suspicious lesions were identified. In the presence of lesions on the oral mucosa, an excisional biopsy was performed. Oral brushing was not performed, as it has low specificity for the detection of HPV-associated precancerous lesions. Indeed, screening based on oral HPV detection is not recommended in low-risk populations [11].

In cases of confirmed HPV-related lesions, patients were enrolled in a follow-up program to monitor the condition of the mucous membranes and to detect any recurrences or new lesions. Patients without lesions were scheduled for annual follow-up.

#### Otorhinolaryngological examination

The otorhinolaryngological evaluation consisted of a meticulous examination of the buccal mucosa and visible oropharyngeal mucosa, with particular emphasis on the tonsils, tongue and soft palate, followed by a systematic inspection of both nasal cavities and the exploration with the fibre optic scope of the rhinopharynx, oropharynx, hypopharynx, and larynx. Each recess, including the glossoepiglottic folds and pyriform sinuses, underwent meticulous analysis for any mucosal anomalies. The evaluation extended beyond the morphological aspects, including a thorough evaluation of the functional aspects and mobility. In instances where suspicious lesions of the pharyngeal, oral, or laryngeal mucosa were identified, a biopsy was promptly scheduled and conducted. Patients without lesions were sent for annual follow-up.

#### Pathology report

Tissue samples obtained during the biopsies from each district were fixed in 10% buffered formaldehyde and sent to the Department of Anatomy and Pathological Histology of the Policlinico Umberto I for histopathological evaluation. All the specimens collected were histologically analysed and routine HPV research, performed through p16 protein analysis, was conducted for every sample.

#### Virological assessment

Cells were centrifuged at low speed, total DNA was purified from cell pellet and used for HPV DNA detection using the Anyplex™ II HPV28 (Seegene Inc., Seoul, Korea) kit that provides simultaneous detection and genotyping of 28 HPV types: 14 types considered at high risk of causing cancer (16, 18, 31, 33, 35, 39, 45, 51, 52, 56, 58, 59, 66, 68) and 14 types considered at low risk of causing cancer (HPV 6, 11, 26, 40, 42, 43, 44, 53, 54, 61, 69, 70, 73, 82). According to the manufacturer, the assay’s limit of detection (sensitivity) was 50 genomic copies per reaction.

### Measure and reference standard

The following variables were collected and analysed as follows: (I) clinical, such as age (years), smoking habit (yes/no), HPV vaccination (yes/no), smoking habit, parity (0/1 +), estrogen-progestin therapy (yes/no), immune suppression (yes/no); (II) cervical, such as HPV test (LR/HR), Pap smear (negative/ASCUS/LSIL/HSIL/Carcinoma in situ), colposcopy (normal/ANTZ1/ANTZ2), cervical histology (CIN1/CIN2/CIN3/Carcinoma in situ; grouped in CIN1/CIN2 +, that included CIN2, CIN3 and Carcinoma in situ, according to the similar management recommended by the guidelines [6]); (III) anal, such as HPV test (negative/positive LR/Positive HR); anal Pap smear (negative/LSIL/HSIL); HRA (absence/presence of visible lesions); histology (negative/LSIL/HSIL); (IV) odontostomatological, such as the examination outcome (absence/presence of visible lesions); and (V) otorhinolaryngological, such as laryngoscopy outcome (absence/presence of visible lesions). Anal coinfection was defined by positivity on aHPV testing, anal brush cytology, or biopsy detection. Odontostomatological and/or otorhinolaryngological coinfections were defined by the presence of histologically confirmed HPV-related lesions in the oral and laryngeal districts, respectively.

### Statistical analysis

Study sample size was based on the calculation proposed by Hajian-Tilaki for the evaluation of diagnostic protocols. According to the Hajian-Tilaki analysis, assuming a sensitivity of 95% in identifying multiple HPV-related lesions and an incidence of multiple lesions of 60%, with the estimation precision (i.e., maximum marginal error) d = 5%, and a type I error alpha = 0.05, a sample of 150 patients is necessary to demonstrate the hypothesis that a multidisciplinary evaluation allows identifying the presence of multiple lesions [12].

Collected data from electronic medical records were entered into an Excel database. Quantitative variables were expressed as median and range. Categorical variables were expressed as counts and percentages. Comparisons between groups were performed using the Kruskal–Wallis test or Fisher’s exact test, as appropriate. Effect sizes were expressed as odds ratios (ORs) with 95% confidence intervals (CIs) or as Cramér’s V, when applicable. A statistical level of 0.05 was used for all the analysis. Statistical software R version 4.0.4 was used for all the analyses.

## Results

In total, 150 women were enrolled in the study. As detailed in Table 1, the median age was 40 years (range 25–77), mostly not smokers (60.7%), nor vaccinated for HPV (57.4), nulliparous (67), no assuming estrogen-progestin therapy (79.7%) and not suffering immunosuppressive condition (91.4%).

**Table 1:** Characteristics of the patients

Median age, years (IQR)	40 (25–77)
Smoking habit (yes), N (%)	59 (39.3%)
HPV vaccination (yes), N (%)	65 (42.6%)
Parity (1 +), N (%)	49 (33%)
Estrogen-Progestin therapy (yes), N (%)	29 (19.3%)
Immune suppression (yes), N (%)	13 (8.6%)

Data expressed as medians and IQR or proportions (percentage) as appropriate

Cervical HR-HPV infection was observed in 69.3% of cases, while LR-HPV infection was observed only in 30.7%. In about half of cases, the results of cervical cytology and colposcopy were ASCUS/LSIL (50.6%) and ANTZ1 (54%), respectively. During colposcopy, biopsies of suspicious lesions were collected in 75 individuals, with a histology report showing CIN2 + in 61.2% of cases (Table 2).

**Table 2:** Outcomes of multidistrict investigation

*Cervical outcomes*	
Cervical HPV test N (%)/total^#^ LR HR	150 (100%)/150 47 (30.7%) 103 (69.3%)
Cervical Pap smear N (%)/total^#^ Normal ASCUS/LSIL HSIL Carcinoma in situ	150 (100%)/150 49 (32.7%) 76 (50.6%) 24 (16%) 1 (0.7%)
Colposcopy N (%)/total^#^ Normal ANTZ1 ANTZ2	150 (100%)/150 43 (29%) 81 (54%) 26 (17%)
Cervical Histology N (%)/total^#^ CIN1 CIN2 CIN3 Carcinoma in situ	75 (100%)/75 29 (38.8%) 20 (26.6%) 19 (25.3%) 7 (9.3%)
*Anal outcomes*	
Anal HPV test N (%)/total^#^ Negative Positive LR Positive HR	150 (100%)/150 73 (48.6%) 30 (20%) 47 (31.3%)
Anal Pap Smear N (%)/total^#^ Negative LSIL HSIL	150 (100%)/150 97 (64.6%) 47 (31.3%) 6 (4%)
High Resolution Anoscopy N (%)/total^#^ Absence of visible lesions Presence of visible lesions	93 (%)/150 29 (31.1%) 64 (68.8%)
Anal Histology N (%)/total^#^ Negative LSIL HSIL	64 (100%)/64 9 (12.5%) 30 (46.9%) 25 (39%)
*Oropharyngeal outcomes*	
Odontostomatological examination N (%)/total^#^ Absence of visible lesions Presence of visible lesions	69 (46%)/150 61 (88.4%) 8 (11.5%)
Laringoscopy N (%)/total^#^ Absence of visible lesions Presence of visible lesions	150 (100%)/150 140 (93.3%) 10 (6.6%)

Data expressed as medians and IQR or proportions (percentage) as appropriate. Abbreviations as described in the text; ^#^ out of the total number of eligible patients

Almost half of the cohort presented anal HPV (aHPV) infection, accounting for 51.3% of women. Among these, the prevalence of HR-aHPV was 31.3%. Conversely anal cytology resulted normally in 64.6% of cases. Ninety- three patients (62%) undergo HRA, according to clinical evaluation and guidelines indications; a biopsy of suspicious lesions was performed in 64 patients (48%), with a result of anal LSIL (aLSIL) and anal HSIL (aHSIL) in 46.9% and 39% of cases, respectively. (Table 2).

About odontostomatological examination, 81 women (54%) refused the examination, while only 46% of patients gave the consent; among these, in 8 patients (11.5%) an oral lesion was detected, removed and confirmed to be HPV-related at the histopathological report (Table 2).

All women performed otorhinolaryngological assessment; only in 10 patients (6.6%) a lesion was detected, removed and confirmed to be HPV- related at the histopathological results (Table 2).

### Concomitant HPV infections

**Fig. 1:**
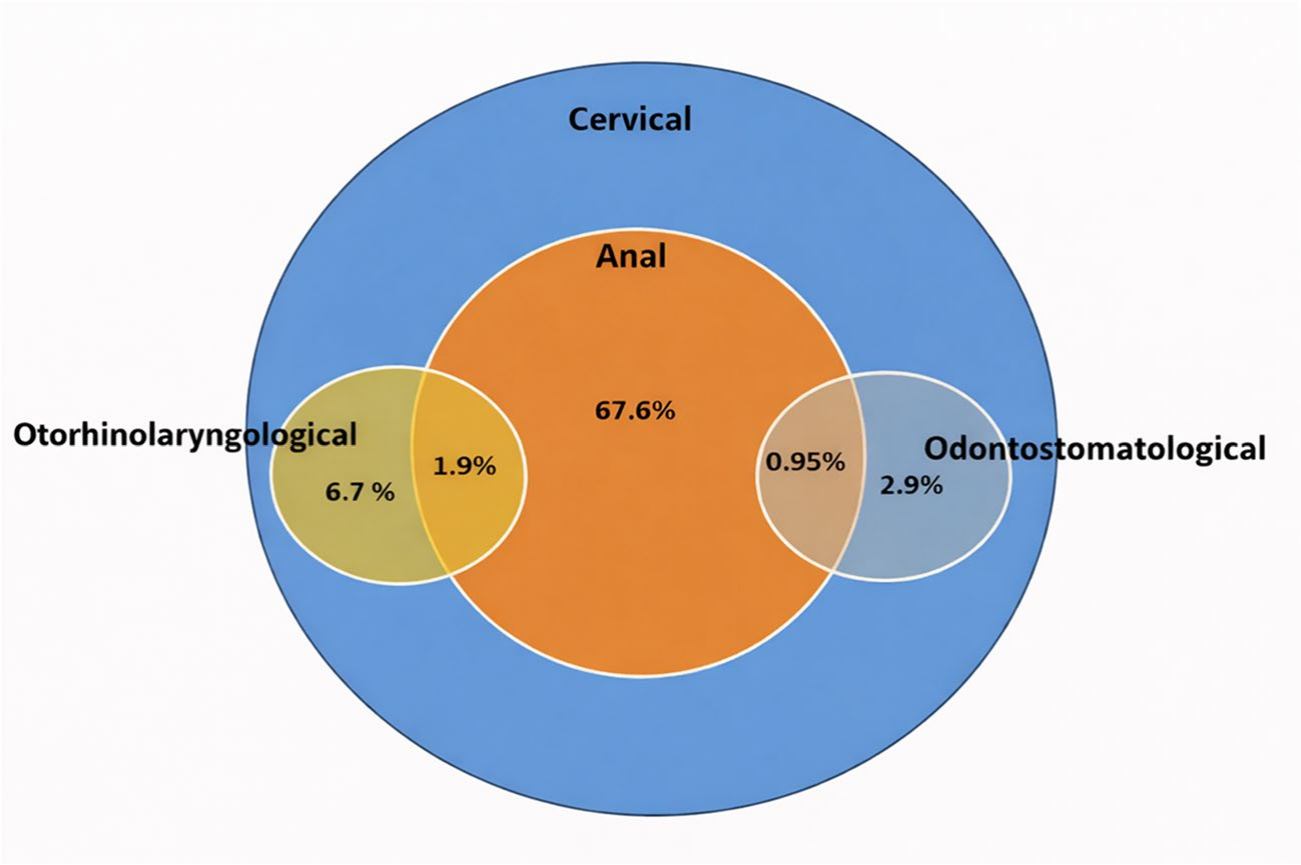
Venn diagram illustrating the prevalence of HPV infection across the investigated sites

As detailed by the Fig. 1, among cHPV positive patients, the final analysis of the multi-districtual screening results, HPV infection was diagnosed in 67%, 6.7%, and 2.9% of anal, otorhinolaryngological, and odontostomatological investigations, respectively. The multiple co-infection was present only in 1.9% in anal, otorhinolaryngological and cervical sites, while in 0.9%, in anal, odontostomatological and cervical sites.

Among the 77 (51.3%) patients with positive aHPV test, 54 patients (70.5%) also had a concomitant cervical HR- HPV infection, while 38 (29.5%) had a cervical LR- HPV; besides, 23 patients (29.6%) had a CIN 2 +.

Among the 54 patients with histologically confirmed anal lesions, 34 patients also had a concomitant cervical HR- HPV infection, while 31 had a cervical LR- HPV. Eleven patients had both LR + HR cHPV infection. Seventeen patients (31.7%) had a CIN2 +.

No statistically significant differences were noted regarding cervical and anal concomitant infection when considering cHPV-genotype (*p* = 0.50; OR: 0.70; CI 95%: 0.29—1.75), cervical PAP-smear results (*p* = 0.67; *V* = 0.13), colposcopy (*p* = 0.14; *V* = 0.19), assumption of estrogen-progestin therapies (*p* = 1; OR: 0.87; CI 95%: 0.30—2.51) and smoking habits (*p* = 0.67; OR: 1.27; CI 95%: 0.54—2.96). In contrast, among patients with aHPV infection, the prevalence of immunosuppressive status was significantly higher (*p* = 0.03), as was the prevalence of CIN2 + on cervical histology (*p* = 0.02; *V* = 0.38). The association between aHPV infection and CIN2 + remained statistically significant after adjustment for age, smoking status, and immunosuppressive condition (*p* = 0.02; OR: 10.5; CI 95%: 0.59—186).

Among the 8 of 69 patients with odontostomatological lesions, five had a positive cervical HR-HPV test with genotype 16/18, and six (75%) had a concomitant cervical lesion (one CIN1, two CIN2 and three CIN 3); in 4 out of 8 patients, a concomitant anal HPV infection was detected. No statistically significant differences were noted regarding cervical and odontostomatological concomitant infection when considering cHPV-genotype (*p* = 1.0; OR: 0.91 CI 95%: 0.08—10.5), cervical PAP-smear results (*p* = 1.0; *V* = 0.08), colposcopy (*p* = 0.42; *V* = 0.12), cervical histology (*p* = 0.73; *V* = 0.15), assumption of estrogen-progestin therapies (*p* = 1.0; OR: 1.74 CI 95%: 0.08—35.0), smoking habits (*p* = 0.56; OR: 3.23 CI 95%: 0.28—36.8) and immunosuppressive status (*p* = 1.0; OR: 1.41 CI 95%: 0.06—29.3).

Among the 10 patients with otorhinolaryngological lesions, 7 (70%) had a cervical HR-HPV infection; in particular 5 were positive for genotypes 16 or 18; in relation to cervical histology, 3 patients presented CIN1, while 4 patients presented concomitant CIN3; in 5 out of 10 patients (50%) there was a positive finding of infection also at the anal level. Three patients (30%) had concomitant lesions also in the oral cavity.

No statistically significant differences were noted regarding cervical and otorhinolaryngological concomitant infection when considering cHPV-genotype (*p* = 0.43; OR: 2.91 CI 95%: 0.33—25.2), cervical PAP-smear results (*p* = 0.41; *V* = 0.13), colposcopy (*p* = 0.26; *V* = 0.18), cervical histology (*p* = 0.05; *V* = 0.18), assumption of estrogen- progestin therapies (*p* = 1.0; OR: 1.44 CI 95%: 0.16—12.7), smoking habits (*p* = 1.0; OR: 1.18 CI 95%: 0.25—5.59) and immunosuppressive status (*p* = 0.48; OR: 1.88 CI 95%: 0.20—17.6).

## Discussion

This study examined, through a combination of molecular tests, cytology, histology, and clinical examination, the prevalence of a concomitant HPV infection in the anogenital and oropharyngeal mucosa in a cohort of women, predominantly immunocompetent, with a positive cHPV-DNA test result.

Previous studies have analysed the concurrent or sequential HPV positivity in the cervical and anal mucosa [13–17] in HIV-positive or other high-risk women, with high rates (30–60%) of concomitant anal infection in cHPV-positive women being reported. A study conducted at our same institution [18] identified the presence of anal HPV DNA in 28% of women attending the Proctology clinic, with approximately half of these cases exhibiting concomitant positivity in the cervical mucosa. Notwithstanding the different methodological approach, these findings are consistent with our own results, indicating that approximately half of low-risk women with positive cHPV results may also harbor anal infection.

Few studies analyzed the HPV positivity in the cervical, anal and oral district in immunocompetent women [2, 19]. In the study by Sehnal et al. [2] conducted among Czech women, concurrent cervical-anal infection was observed in 27.1% of women diagnosed with a CIN2 + and in 10.7% of women with negative cervical histology or CIN1, whereas the cervix-oral concurrent HPV positivity was far lower (1.7% and 0.7% in the CIN2 + and in the negative/CIN1 groups, respectively). Accordingly, data coming from the present study confirm that patients with CIN2 + might be at greater risk for anal dysplasia and, therefore, could benefit from anal screening. Having a CIN2 + was significantly associated with anal coinfection. Although no significant confounding effects from age or immunosuppression were observed, these exploratory findings suggest that anal site screening may warrant consideration in patients with HPV-related cervical lesions, pending confirmation in larger studies.

In a recent Italian study [19] involving 81 women attending a Sexual Transmitted Infections Clinic (cases) and age-matched women attending the Dermatology unit (controls), the concurrent HPV positivity in the cervical and anal samples was 45%. Differently from our results, this study detected a far higher oral HPV positivity, detected by collecting brushing from the oral cavity: 29% oral HPV infection detected in women with cHPV infection and in 40% of the women with anal HPV, but the oral-cervical and oral-anal HPV infection were not statistically associated. In the present study we did not perform oral brushing to detect HPV since there are not clear guidelines on how to collect samples [20]. Considering the different study designs, the rates of concurrent cervical and anal HPV positivity are comparable with those observed in our cohort. However, differences in patient selection should be considered. In particular, in our study, all patients were already attending a colposcopy outpatient clinic for a cHPV proven infection, whereas in the similar study by Senhal et al., patients were recruited regardless of cervical HPV positivity, indeed 18.1% were HPV-negative at all investigated sites. This difference in baseline HPV prevalence may partly explain the slightly higher prevalence of anal coinfection observed among CIN2 + patients in our study (30.5% vs. 27.1%).

Cervical determinants for the risk of harbouring anal precancerous lesions comprehend cervical HSIL and cervical HR-HPV infection, with a higher risk in the case of HPV 16 infection [21]. Female population subgroups with higher risk of squamous cell carcinoma of the anus have been identified and recent guidelines suggest when and how to screen individuals with an increased risk of this neoplasm [22]. In particular, current consensus guidelines for anal cancer screening [23] consider absolute indications to anal screening in: HIV patients, men having sex with men, women with a history of high grade vulvar intraepithelial lesion or vulvar cancer within 1 year of diagnosis, solid organs transplant recipients; relative indications to anal screening are: cervical or vaginal high grade intraepithelial lesion or cancers in patients > 45 years old with anal symptoms, visible lesions in perianal region, persistent cHPV 16 (> 1 year), other immunosuppression statuses. Our findings support current guidelines, as women with immunosuppression showed a higher likelihood of having concomitant aHPV infection.

As to oral HPV coinfection data are conflicting since the low prevalence of these diseases. In the present study we detected oral lesions in 8 of 69 (11.5%) and laryngeal lesions in 10 of 150 (6.6%) patients, all benign.

However multi-site HPV infection warrants more in-depth screening beyond the anal site in select vulnerable subgroups, such as immunocompromised patients, and standardized assessments of head-and-neck regions should be studied and included in dedicated diagnostic-therapeutic pathways. Indeed, these patients show a higher prevalence of multi-site infections, high-grade lesions, and HPV-related head-and-neck lesions, despite their relatively low incidence in the general population.

In addition to these issues, to date several aspects of multisite infection remain unknown and unexplored. Indeed, although some well-known risk factors, such as the presence of HR genotypes or immunosuppressive conditions may affect individual susceptibility, they do not fully explain the clinical spread of persistent infection or the consequent oncogenic potential of the virus. Moreover, although HPV transmission is known to occur through mucosal surfaces, few studies have focused on the role of concomitant mucosal microbiota or on impairments of the local immune system [23].

Considering all these aspects, promoting adherence to global HPV vaccination programs will become increasingly important. Vaccination will play a central role, influencing future screening strategies adapted to improved coverage rates among younger populations, and employed as adjuvant therapy for patients undergoing excisional treatment of HPV related lesions of all the anatomical districts [24–26].

Our study is inherently limited by several factors. First, methodological heterogeneity across the investigated sites, with different diagnostic work-up, may have affected the study findings. In particular, brush-based HPV testing in the oral and otorhinolaryngological districts, as performed in this study, is neither standardized nor recommended by current guidelines. These limitations highlight the need for future and standardized multidisciplinary guidelines.

Second, the investigation of the different districts was restricted to a single-time-point analysis of the presence of HPV and/or clinical lesions; consequently, it is not possible to directly evaluate whether the persistence of cervical HPV infections leads to an increased risk of anal/oral infection. In addition, findings related to HPV lesions identified by HRA should be interpreted with caution, as they may be affected by selection bias. Specifically, HRA was performed in accordance with current guidelines only in women with a positive aHPV test or an abnormal anal Pap smear, rather than in all patients with cHPV included in the study.

Third, the limited sample size of this study precluded a robust evaluation of the differential impact of cHPV infection on the oropharyngeal district. In particular, the high rate of refusal of the odontostomatological examination further limits the reliability and generalizability of the related findings; from another perspective, however, this also highlights the practical difficulties in implementing diagnostic work-up in these sites, potentially reducing its overall accuracy.

Immunosuppressed individuals are recognized to be at higher risk of HPV coinfections and HPV-related diseases. In this context, more comprehensive diagnostic strategies may be considered, with particular attention to approaches that could help overcome patient-related barriers to adherence.

## Conclusions

In women with cHPV infection, even those who are immunocompetent, the risk of concurrent HPV infection at multiple anatomical sites, including the anal, oral and otorhinolaryngological regions, appears to be highly variable. Among these, aHPV infection is particularly noteworthy. The findings of this study suggest that the presence of a cervical lesion classified as CIN2 or higher may be associated with a higher likelihood of aHPV coinfection. Accordingly, aHPV assessment, such as anal brushing, could be considered, particularly in comparison with the evaluation of oral and laryngeal sites. However, given the exploratory nature of the study and its limited sample size, these results should be interpreted with caution and cannot be readily generalized. Validation in larger, multicentric, longitudinal studies is required before these observations can inform future multidisciplinary guidelines. Such guidelines are urgently needed to define a consistent and evidence-based approach to the diagnosis and management of HPV infection, which is currently fragmented and inconsistent.

## Data Availability

Database generated during and/or analyzed during the current study is available from the corresponding author on reasonable request.

## References

[1] Dunne EF et al (2007) Prevalence of HPV infection among females in the United States. JAMA 297(8):813. 10.1001/jama.297.8.813 17327523 10.1001/jama.297.8.813

[2] Sehnal B et al (2019) The association among cervical, anal, and oral HPV infections in high-risk and low-risk women. Eur J Obstet Gynecol Reprod Biol: X 4:100061. 10.1016/j.eurox.2019.100061 31517298 10.1016/j.eurox.2019.100061 PMC6728742

[3] Sung H et al (2021) Global cancer statistics 2020: GLOBOCAN estimates of incidence and mortality worldwide for 36 cancers in 185 countries. CA Cancer J Clin 71:209–249. 10.3322/caac.21660 33538338 10.3322/caac.21660

[4] Forman D et al (2012) Global burden of human papillomavirus and related diseases. Vaccine 30:F12–F23. 10.1016/j.vaccine.2012.07.055 23199955 10.1016/j.vaccine.2012.07.055

[5] Markowitz LE et al (2020) Human papillomavirus vaccine effectiveness against HPV infection: evaluation of one, two, and three doses. J Infect Dis 221(6):910–918. 10.1093/infdis/jiz555 31784749 10.1093/infdis/jiz555 PMC13137874

[6] Kyrgiou M et al (2020) Cervical screening: ESGO-EFC position paper of the European Society of Gynaecologic Oncology (ESGO) and the European Federation of Colposcopy (EFC). Br J Cancer 123(4):510–517. 10.1038/s41416-020-0920-9 32507855 10.1038/s41416-020-0920-9 PMC7434873

[7] Nayar R (2015) The Bethesda system for reporting cervical cytology, 3rd edn. Springer, Cham

[8] International Agency for Research on Cancer (2020) WHO classification of tumours: female genital tumours, vol vol 4, 5th ed. IARC, Lyon

[9] Darragh TM et al (2012) The lower anogenital squamous terminology standardization project for HPV-associated lesions: Background and consensus recommendations from the College of American Pathologists and the American Society for Colposcopy and Cervical Pathology. Arch Pathol Lab Med 136(10):1266–1297. 10.5858/arpaLGT200570 22742517 10.5858/arpa.LGT200570

[10] Hillman RJ et al (2016) 2016 IANS international guidelines for practice standards in the detection of anal cancer precursors. J Low Genit Tract Dis 20(4):283–291. 10.1097/LGT.0000000000000256 27561134 10.1097/LGT.0000000000000256

[11] D’Souza G et al (2017) Understanding personal risk of oropharyngeal cancer: risk-groups for oncogenic oral HPV infection and oropharyngeal cancer. Ann Oncol 28(12):3065–3069. 10.1093/annonc/mdx535 29059337 10.1093/annonc/mdx535 PMC5834136

[12] Hajian-Tilaki K (2014) Sample size estimation in diagnostic test studies of biomedical informatics. J Biomed Inform 48:193–204. 10.1016/j.jbi.2014.02.013 24582925 10.1016/j.jbi.2014.02.013

[13] Kojic EM et al (2011) Human papillomavirus infection and cytologic abnormalities of the anus and cervix among HIV-infected women in the study to understand the natural history of HIV/AIDS in the era of effective therapy (the SUN study). Sex Transm Dis 38(4):253–259. 10.1097/OLQ.0b013e3181f70253 20966828 10.1097/OLQ.0b013e3181f70253

[14] de Pokomandy A et al (2017) The EVVA cohort study: anal and cervical type-specific human papillomavirus prevalence, persistence, and cytologic findings in women living with HIV. J Infect Dis 216(4):447–456. 10.1093/infdis/jix273 28931234 10.1093/infdis/jix273

[15] Goodman MT et al (2008) Acquisition of anal human papillomavirus (HPV) infection in women: the Hawaii HPV cohort study. J Infect Dis 197(7):957–966. 10.1086/529207 18429348 10.1086/529207

[16] Shvetsov YB et al (2009) Duration and clearance of anal human papillomavirus (HPV) infection among women: the Hawaii HPV cohort study. Clin Infect Dis 48(5):536–546. 10.1086/596758 19191636 10.1086/596758 PMC2756215

[17] Palefsky JM et al (2001) Prevalence and risk factors for anal human papillomavirus infection in human immunodeficiency virus (HIV)–positive and high‐risk HIV‐negative women. J Infect Dis 183(3):383–391. 10.1086/318071 11133369 10.1086/318071

[18] Pierangeli A et al (2012) High detection rate of human papillomavirus in anal brushings from women attending a proctology clinic. J Infect 65(3):255–261. 10.1016/j.jinf.2012.05.004 22609230 10.1016/j.jinf.2012.05.004

[19] Herzum A et al (2022) Cervical, oral and anal human papillomavirus infection in women attending the dermatology unit of a regional reference hospital in Genoa, Italy: a prevalence study. J Prev Med Hyg 63(3):E415–E419. 10.15167/2421-4248/jpmh2022.63.3.1697 36415298 10.15167/2421-4248/jpmh2022.63.3.1697 PMC9648552

[20] Poljak M et al (2023) Testing for human papillomaviruses in urine, blood, and oral specimens: an update for the laboratory. J Clin Microbiol 61(8):e0140322. 10.1128/jcm.01403-22 37439692 10.1128/jcm.01403-22 PMC10446865

[21] Lin C et al (2019) Cervical determinants of anal HPV infection and high-grade anal lesions in women: a collaborative pooled analysis. Lancet Infect Dis 19(8):880–891. 10.1016/S1473-3099(19)30164-1 31204304 10.1016/S1473-3099(19)30164-1 PMC6656696

[22] Stier EA et al (2024) International anal neoplasia society’s consensus guidelines for anal cancer screening. Int J Cancer 154(10):1694–1702. 10.1002/ijc.34850 38297406 10.1002/ijc.34850

[23] Dong M et al (2023) Interactions between microbiota and cervical epithelial, immune, and mucus barrier. Front Cell Infect Microbiol 13:1124591. 10.3389/fcimb.2023.1124591 36909729 10.3389/fcimb.2023.1124591 PMC9998931

[24] Petráš M et al (2023) Timing of HPV vaccination as adjuvant treatment of CIN2+ recurrence in women undergoing surgical excision: a meta-analysis and meta-regression. Sex Transm Infect 99(8):561–570. 10.1136/sextrans-2023-055793 37553234 10.1136/sextrans-2023-055793 PMC10715477

[25] Deshmukh AA et al (2017) Adjuvant HPV vaccination for anal cancer prevention in HIV-positive men who have sex with men: the time is now. Vaccine 35(38):5102–5109. 10.1016/j.vaccine.2017.08.006 28807605 10.1016/j.vaccine.2017.08.006 PMC5581672

[26] Di Donato V et al (2022) HPV vaccination after primary treatment of HPV-related disease across different organ sites: a multidisciplinary comprehensive review and meta-analysis. Vaccines 10(2):239. 10.3390/vaccines10020239 35214697 10.3390/vaccines10020239 PMC8879645

